# Polyphenolic Platform Ameliorated Sanshool for Skin Photoprotection

**DOI:** 10.1002/advs.202310012

**Published:** 2024-02-15

**Authors:** Tianyou Wang, Linghong Guo, Shuwei Wu, Yuanyuan Xu, Junmei Song, Yi Yang, Hengjie Zhang, Dongcui Li, Yiwen Li, Xian Jiang, Zhipeng Gu

**Affiliations:** ^1^ College of Polymer Science and Engineering State Key Laboratory of Polymer Materials Engineering Sichuan University Chengdu 610065 China; ^2^ Department of Dermatology West China Hospital, Sichuan University Chengdu 610041 China; ^3^ Laboratory of Dermatology Clinical Institute of Inflammation and Immunology Frontiers Science Center for Disease‐related Molecular Network West China Hospital, Sichuan University Chengdu 610041 China; ^4^ Hua An Tang Biotech Group Co., Ltd. Guangzhou 511434 China

**Keywords:** free radical scavenging, natural polyphenols, photodamage, sanshool, self‐assembly

## Abstract

Natural evolution has nurtured a series of active molecules that play vital roles in physiological systems, but their further applications have been severely limited by rapid deactivation, short cycle time, and potential toxicity after isolation. For instance, the instability of structures and properties has greatly descended when sanshool is derived from *Zanthoxylum xanthoxylum*. Herein, natural polyphenols are employed to boost the key properties of sanshool by fabricating a series of nanoparticles (NPs). The intracellular evaluation and in vivo animal model are conducted to demonstrate the decreased photodamage score and skin‐fold thickness of prepared NPs, which can be attributed to the better biocompatibility, improved free radical scavenging, down‐regulated apoptosis ratios, and reduced DNA double‐strand breaks compared to naked sanshool. This work proposes a novel strategy to boost the key properties of naturally occurring active molecules with the assistance of natural polyphenol‐based platforms.

## Graphical abstract

A polyphenolic platform is utilized to ameliorate the anti‐oxidative and anti‐ultraviolet stability of sanshool *Zanthoxylum xanthoxylum*. The protective effects against photodamage are confirmed with intracellular and in vivo models by improved stability, down‐regulated apoptosis, and reduced DNA breaks compared to naked sanshool. It proposed a novel strategy to boost the key properties of natural active molecules with polyphenolic platforms.

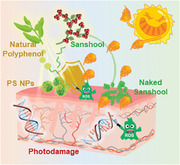

## 1 Introduction

Natural evolution has yielded a diverse array of functionally active natural products, including vitamins, alkaloids, and neurotransmitters.^[^ ^1^, ^2^ ^]^ These natural products possess specific physiological properties such as signal transduction, free radical scavenging, and photoprotection, which enable them to overcome the challenges of complex environments.^[^ ^3^, ^4^, ^5^, ^6^, ^7^ ^]^ For instance, hydroxy‐α‐sanshool derived from *Zanthoxylum xanthoxylum* has garnered significant interest in skin care applications due to its classical conjugated long fatty chain, which imparts excellent properties such as scavenging reactive oxygen species (ROS) and absorbing UV irradiation.^[^ ^6^ ^]^ Consequently, previous works have been conducted to demonstrate the photoprotective effects of sanshool against UV‐induced photodamage and its potential for improving crow's feet therapy via down‐regulating intracellular ROS levels.^[^ ^6^, ^8^ ^]^ However, the further advancement of highly active molecules is hindered by their rapid deactivation, short cycle time, and potential toxicity.^[^ ^9^, ^10^, ^11^, ^12^ ^]^ Despite the immense potential of sanshool in biomedical fields, its inherent instabilities and insufficient bioavailability restrict its applications in more demanding contexts. Especially, the deactivation reactions triggered by sunlight irradiation would significantly diminish the photo absorbance and ROS scavenging capabilities, which are crucial for photoprotection.^[^ ^10^ ^]^ Therefore, rational designs should be proposed to stabilize the active structures of sanshool and further promote the key properties and stability of sanshool for further applications against photodamage.

Natural polyphenols, a group of naturally occurring active molecules primarily found in plants, have been extensively investigated in recent time and confirmed to possess excellent biocompatibility as well as various physiological activities including antioxidative abilities, vascular protection, and regulation of inflammation.^[^ ^13^, ^14^, ^15^, ^16^, ^17^, ^18^ ^]^ Moreover, the chemical structures of polyphenols have garnered significant attention due to the presence of interesting catechol and pyrogallol groups.^[^ ^19^ ^]^ They could interact with each other or other functional molecules through hydrogen bonding, electrostatic interactions, and polymerization, enabling them to serve as both functors and synthons.^[^ ^20^, ^21^, ^22^, ^23^ ^]^ Based on the structural and functional properties, natural polyphenols‐based materials have been fabricated and applied to address key challenges in numerous fields.^[^ ^24^, ^25^, ^26^, ^27^, ^28^, ^29^, ^30^, ^31^, ^32^, ^33^ ^]^ Notably, it could also serve as an effective platform to boost the key properties of functional materials based on the features of both structures and functions.^[^ ^34^, ^35^, ^36^, ^37^ ^]^ Therefore, it could be speculated that natural polyphenols could be employed as valuable building blocks to boost the key properties of sanshool against photodamage via reasonable integration strategies.

In this study, a series of polyphenol‐sanshool (PS) nanoparticles (NPs) have been successfully fabricated via the boron ester reactions and noncovalent interactions (mainly including hydrophobic and hydrogen bond interactions) between natural polyphenols and phenylboronic acid group modified sanshool. The epigallocatechin gallate (EGCG), tea polyphenols, and grape seed polyphenols, which have sufficient catechol structures and good water solubility, were selected as representative examples, and yielding NPs with uniform size were labeled as ES, TS, and GS NPs, respectively. Moreover, the key properties and corresponding stabilities, such as photo absorbance and antioxidative abilities, were investigated, and all NPs were carefully investigated to exhibit improved stability with the assistance of polyphenols. Subsequently, intracellular, and in vivo experiments demonstrated excellent biocompatibility, down‐regulated levels of ROS, apoptosis, and photodamage‐induced genotoxicity with the treatment of PS NPs. Interestingly, the NPs outperformed naked sanshool in both intracellular and in vivo experiments, which could be attributed to the boosted effects of natural polyphenols. Overall, it provided a general and robust strategy to boost the key properties of natural active molecules with polyphenolic platform.

## 2 Results and Discussion

### 2.1 Preparation and Characterization of NPs

**Figure 1:**
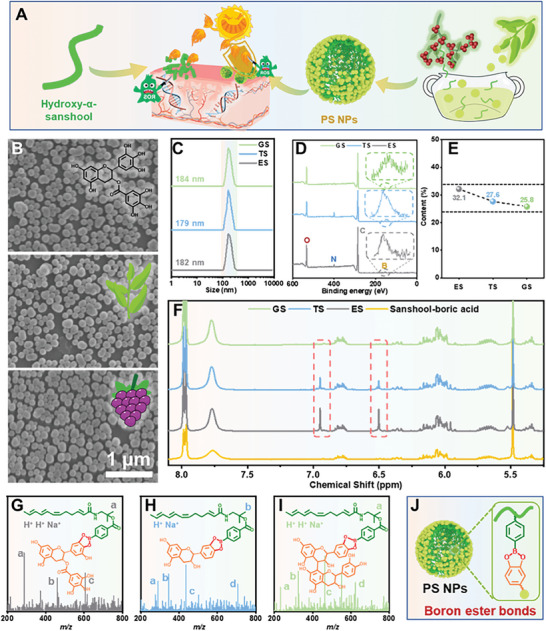
Preparation and characterization of PS NPs. A) The schematic illustration of PS NPs fabrication processes. B) The SEM images, C) DLS results, and D) XPS spectra of ES, TS, and GS NPs, respectively. E) The sanshool contents in each NPs, respectively. F) The ^1^H NMR spectra of disassembled NPs including ES, TS, GS NPs as well as sanshool‐boric acid. The ESI‐MS spectra and proposed structures of G) ES, H) TS, and I) GS NPs, respectively. J) The schematic illustration of PS NPs structures.

Herein, a series of PS NPs have been successfully fabricated with the active sanshool molecules from *Zanthoxylum xanthoxylum* and plants (**Figure** **1**A). Initially, phenylboronic acid group modified sanshool from *Zanthoxylum xanthoxylum* was first synthesized (Figure S1, Supporting Information) and then combined with a range of polyphenols (including EGCG, tea polyphenols, and grape seed polyphenols) found in plants through multiple intramolecular interactions. The resulting NPs were respectively labeled as ES, TS, and GS NPs, which were confirmed with scanning electron microscope (SEM) and dynamic light scattering (DLS) assays (Figure 1B,C). The fabricated NPs exhibited uniform morphologies, similar sizes ≈140 nm, and narrow dispersion (Figure 1B,C). The negative zeta potential ensured sufficient stabilities for further applications (Figure S2, Supporting Information). Furthermore, the structures and formation mechanism of PS NPs were investigated. The X‐ray photoelectron spectroscopy (XPS) spectra of the NPs revealed the presence of the phenylboronic acid group‐modified sanshool by detecting B and N elements that were not existing in selected polyphenols (Figure 1D). The quantitative results of the above elements could also determine the content of sanshool in each NPs. The ES NPs exhibited the highest doping content, likely due to the presence of more catechol structures for boron ester formation and enhanced intermolecular interactions (Figure 1E). The efficient combination between sanshool and polyphenols could also be demonstrated with the ^1^H NMR spectra of NPs disassembled product in deuterated dimethyl sulfoxide (Figure 1F). Then, electrospray ionization mass spectrometry (ESI‐MS) spectra were conducted to analyze the chemical structures of disassembled NPs, and the proposed structures were respectively assigned to the corresponding peaks. The modification of phenylboronic acid group towards sanshool could effectively react with the catechol groups of polyphenols via the formation of boron ester bonds, and the polyphenols could even integrate more than one sanshool which could provide more driving force for subsequent self‐assembly (Figure 1G–J; Figure S3, Supporting Information). Significantly, the proposed structures of NPs could perfectly match up with the elemental fine spectra obtained from XPS analysis (Figure S4, Supporting Information). Thus, the PS NPs with uniform sizes have been prepared via the boron ester formation and following self‐assembly processes, and the properties of NPs will be further evaluated.

### 2.2 Anti‐Ultraviolet and Anti‐Oxidative Properties of NPs

**Figure 2:**
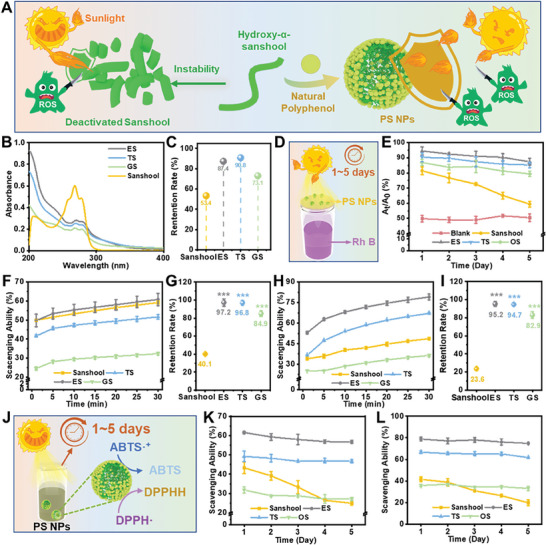
The anti‐ultraviolet and anti‐oxidative properties of PS NPs. A) The schematic illustration of PS NPs with enhanced anti‐ultraviolet and anti‐oxidative properties. B) The UV‐vis spectra of ES, TS, and GS NPs, respectively. C) The absorbance retention rates of sanshool, ES, TS, and GS NPs after 2 h simulated sunlight irradiation. D) The schematic diagram of long‐term photoprotection of PS NPs. E) The photoprotective effects towards Rh B of blank, sanshool, ES, TS, and GS NPs, respectively. The F) scavenging abilities and G) stabilities of sanshool, ES, TS, and GS NPs towards DPPH free radicals. The H) scavenging abilities and I) stabilities of sanshool, ES, TS, and GS NPs towards ABTS free radicals. J) The schematic diagram of long‐term antioxidative stabilities of PS NPs. The long‐term antioxidative stabilities of sanshool, ES, TS, and GS NPs towards K) DPPH and L) ABTS free radicals. The ns represents no significant difference; ^*^ represents *p* < 0.05; ^**^ represents *p* < 0.01; ^***^ represents *p* < 0.001; and ^****^ represents *p* < 0.0001 versus the sanshool group.

The ability to resist ultraviolet in sunlight and subsequent oxidative stress plays a vital role in protecting skin against photodamage.^[^ ^27^, ^38^ ^]^ Therefore, the protective effects and stabilities of PS NPs against sun irradiation were detected in detail (**Figure** **2**A). The UV–vis spectra of prefabricated NPs were first performed and obvious absorbance across the entire UV regions could be observed especially the UVB region which occupies the main part in sunlight (Figure 2B). The absorbance abilities of naked sanshool significantly decreased to only ≈50% after sunlight irradiation which heavily impeded the further application for photoprotection, but all NPs showed enhanced photostability with the assistance of natural polyphenols (Figure 2C). Especially, the ES and TS NPs could retain ≈90% absorbance abilities providing more potential for further applications. Furthermore, a facile model was established to evaluate the photoprotective effects of NPs deposited filter papers with rhodamine B (Rh B, Figure 2D). Without NPs loading, only ≈50% Rh B could be reserved after 2 h simulated sunlight irradiation. Interestingly, the PS NPs containing groups could retain about 90% Rh B after irradiation which was attributed to the excellent UV absorbance effects (Figure 2E). To further evaluate the long‐term photoprotective stabilities, the retention ratios of Rh B were tested with the NPs loaded filter papers stored at room conditions for up to 5 days. Even after 5 days, all PS NPs groups still protected >80% of Rh B (Figure 2E). As a comparison, the naked sanshool could achieve a protective ratio of ≈81.43% after one day, but the ability severely decreased to only 59.41% after 5 days of storage (Figure 2E; Figure S5, Supporting Information). The enhanced long‐term photoprotective effects were assigned to the improved stabilities of PS NPs. After that, the free radical scavenging abilities were subsequently evaluated which also played a critical role in photoprotection. The typical 2,2‐diphenyl‐1‐picrylhydrazyl (DPPH) assay was first conducted, and both naked sanshool, as well as PS NPs, showed excellent scavenging efficacy towards DPPH free radicals (Figure 2F). However, after 2 h simulated sunlight irradiation, the scavenging ability of sanshool rapidly decreased due to the unstable structure, and the scavenging ratios were lower than all PS NPs (Figure 2G; Figure S6, Supporting Information). After that, 2,2‐azino‐bis (3‐ethylbenzothiazoline‐6‐sulfonic acid) (ABTS) assay was also employed to evaluate the scavenging abilities in aqueous solution, and more significant improvements were obtained due to the enhanced water dispersion and colloidal stability of PS NPs (Figure 2H,I; Figure S7, Supporting Information). Noteworthily, ES NPs performed the best free radical scavenging abilities in both assays and photostability, giving them great potential for photoprotection. Moreover, the PS NPs were placed in the outdoor environment for varying durations of up to 5 days, and the free radical scavenging abilities at different time points were detected to assess the long‐term antioxidative stabilities (Figure 2J). Over time, the less free radicals could be scavenged with naked sanshool in both assays, and it could only maintain less than 60% of antioxidative capacity after 5 days (Figure 2K,L; Figures S8–S9, Supporting Information). However, no obvious weakness could be observed in any of the PS NPs during the 5 days, indicating that fabricated PS NPs were endowed with enhanced antioxidative stabilities and huge potential for oxidative stress remission (Figure 2K,L; Figures S8 and S9, Supporting Information). Together, the photoprotective and antioxidative stabilities of sanshool have been roundly improved with the introduction of natural polyphenols, and this strategy could propose a novel platform for the vital performance enhancement of natural active molecules.

### 2.3 Intracellular Photoprotective Effects of NPs Against Photodamage

**Figure 3:**
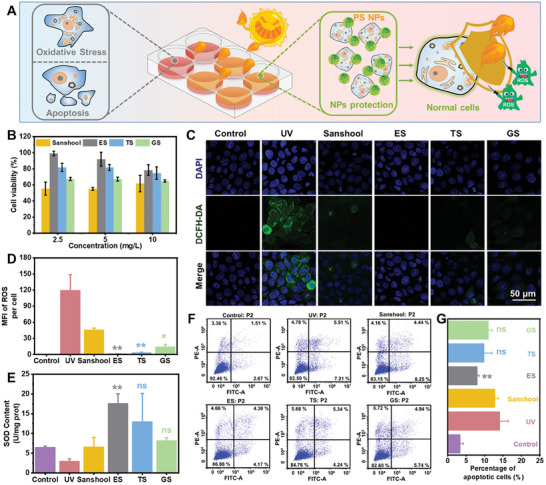
The intracellular photoprotective effects of NPs against photodamage. A) The schematic illustration of intracellular photoprotective effects of PS NPs against photodamage. B) The photodamaged cell viabilities are treated with sanshool, ES, TS, and GS NPs, respectively. C) The DAPI, DCFH‐DA, and merge staining images of HaCaT cells with different treatments. D) Flow cytometry analysis of ROS and E) SOD levels in each group. The F) flow cytometry analysis and G) quantitative results of cell apoptosis (including early and late apoptotic cells). The ns represents no significant difference; ^*^ represents *p* < 0.05; ^**^ represents *p* < 0.01; ^***^ represents *p* < 0.001; and ^****^ represents *p* < 0.0001 versus the sanshool group.

Moreover, we investigated the photoprotective effects of PS NPs at the cellular level (**Figure** **3**A). Prior to assessing their potential biological applications, we initially conducted biocompatibility tests on various PS NPs. The viability of HaCaT cells was evaluated using the cell counting kit‐8 (CCK‐8) assay after pretreatment with PS NPs at concentrations ranging from 2.5 to 40 mg L^−1^ (Figure S10, Supporting Information). The results demonstrated that ES, TS, and GS NPs exhibited better cell compatibility than sanshool at concentrations of 2.5, 5, and 10 mg L^−1^, with cell viability of HaCaT cells exceeding 80%. Subsequently, HaCaT cells were pre‐treated with ES, TS, GS, and sanshool at concentrations of 2.5–10 mg L^−1^, followed by UV exposure (Figure 3B). The results of the CCK‐8 assay showed that PS NPs exhibited significantly better photoprotective effects than sanshool at a concentration of 2.5 mg L^−1^. Therefore, this concentration was chosen for subsequent intracellular experiments. Among them, ES NPs exhibited the best cellular photoprotective effect, with HaCaT cell viability remaining above 95% after exposure to UV. Under extensive UV stimulation, excessive production of ROS in the body and reduced clearance capacity could trigger oxidative stress reactions, directly leading to tissue damage. To further investigate the intracellular free radical scavenging ability of PS NPs, 2,7‐dichlorodihydrofluorescein diacetate (DCFH‐DA) was applied as an indicator to evaluate ROS levels (Figure 3C). Under confocal microscopy, we clearly observed that UV radiation led to increasing ROS levels in HaCaT cells, while treatment with PS NPs resulted in a significant reduction in intracellular ROS expression levels. According to the fluorescence statistics within cells, the mean fluorescence intensity (MFI) of ROS in HaCaT cells significantly increased after UV exposure, but decreased significantly in HaCaT cells treated with PS NPs compared to the sanshool group, with ES exhibiting the lowest ROS expression fluorescence (Figure 3D). We further examined the level of superoxide dismutase (SOD) within the cells to evaluate oxidative damage. As shown in the Figure 3E, although all the PS NPs treated groups exhibited higher SOD expression levels than the sanshool‐treated group, only pre‐treatment with ES NPs showed a statistically significant increase in SOD production, indicating its greater reduction of cell oxidative damage caused by UV radiation. Additionally, another significant impact of oxidative stress on cells is the mediation of cell apoptosis.^[^ ^39^ ^]^ To investigate this, we conducted flow cytometry analysis to detect cell apoptosis. The results showed that the overall apoptosis of HaCaT cells was markedly increased after UV irradiation (Figure 3F,G). In contrast, treatment with PS NPs decreased the level of apoptosis in photodamaged HaCaT cells. Compared to the group treated with sanshool, only the treatment with ES NPs significantly reduced the total number of apoptotic cells (Figure 3F,G). This effect of ES NPs in attenuating apoptosis from photodamage was consistent with the observed promotion in cell viability. The better performance of ES NPs was attributed to the greater photo absorbance and antioxidative abilities, as well as the exceptional stabilities compared to other NPs. The aforementioned results indicate that PS NPs exhibit superior cellular photoprotective effects compared to sanshool, with ES NPs being the most effective.

### 2.4 In Vivo Assessment of Skin Photoprotection

**Figure 4:**
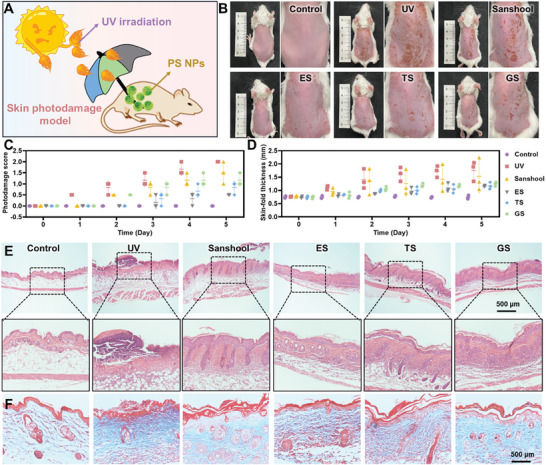
In vivo assessment of skin photoprotective effects. A) The schematic illustration of PS NPs for skin photoprotection. B) The optical images of skin with different treatments after UV irradiation. The C) photodamage score and D) skin‐fold thickness of mice skin with different treatments. The E) H&E and F) Masson staining images of mice skin in each group.

To determine the therapeutic outcomes of PS NPs, a mouse model of skin photodamage was performed to evaluate relevant efficacy indicators in vivo (**Figure** **4**A). Prior to daily UV exposure, blank solvent, sanshool, ES, TS, and GS NPs were evenly applied to the back skin of mice (20 mg kg^−1^, 1.5 cm × 2 cm). Subsequently, UVB irradiation was administered for 5 consecutive days (120 mj cm^−2^ day^−1^), and these mice were sacrificed on the 5th day after irradiation. Mice that did not undergo any treatment or UV exposure were labeled as the control group.

Representative skin photographs from each group were taken daily and compiled in Figure 4B and Figure S11 (Supporting Information), representing the successful establishment of the animal model. The photographs of mouse back skin after the final irradiation were organized in Figure 4B and Figure S11 (Supporting Information), which revealed that the mice's skin in UV group exhibited typical manifestations of skin photodamage, including erythema, roughness, thickening, scab formation, and pigmentation.^[^ ^40^ ^]^ Local application of sanshool partially relieved UV‐induced skin damage, while the overall condition of the mouse back skin treated with PS NPs was better than that of the sanshool group. Based solely on the appearance of the skin, the mouse skin treated with PS NPs showed a trend of superior condition in ES group compared to TS group and further superior to GS group which was consistent with the in vitro photo absorbance and free radical scavenging performance. Furthermore, analysis of the mouse skin damage score revealed that as the cumulative UV radiation increased, the skin damage score of the UV group gradually increased (Figure 4C). In the sanshool‐treated group, the skin damage score increased starting from the 2nd day of UV irradiation, but the final score was still lower than that of the UV group. It is worth noting that the skin damage scores of the ES and TS groups only began to rise slowly from the 3rd day of UV exposure, and the final scores of both groups were significantly lower than those of the sanshool group. Similarly, in the results of skin thickness measurement, the skin thickness of the ES and TS groups showed a gradual increase trend, and the final thickness was lower than that of the sanshool group's back skin (Figure 4D). Since cumulative oxidative stress induced by UV radiation leads to thickening of the stratum corneum and increased disorder and thickening of collagen fibers in the dermis, the skin thickness tended to increase.^[^ ^41^, ^42^ ^]^ This indicates that ES and TS treatments exhibited more stable and superior protective effects against skin photodamage compared to sanshool.

In histopathology, we initially employed hematoxylin‐eosin (H&E) staining to observe the morphological characteristics of the skin tissues on the dorsal region of all mice (Figure 4E). It was evident that the epidermal and dermal layers of the mouse skin exposed to cumulative UV radiation showed significant thickening, as well as a reduction in the natural curvature of the epidermal layer. These observations corresponded to the rough and thickened appearance of the UV‐damaged mouse skin. Treatment with ES NPs significantly rescued the skin condition of the mice, resulting in skin thickness and overall morphology approaching that of the control group. Masson staining was subsequently used to assess the morphology and expression of collagen fibers in the dermal layer of the mouse skin (Figure 4F). UV irradiation caused an increase in thickness, density, and denaturation of the collagen fibers in the mouse skin, even leading to a homogeneous appearance. However, ES NPs demonstrated a remarkable ability to counteract the UV‐induced degeneration of collagen fibers. Specifically, the mice treated with ES exhibited well‐organized and evenly arranged collagen fibers in the skin, with a loose matrix and normal fiber morphology. Similarly, the observed morphology of elastic fibers through Elastin van Gieson (EVG) staining showed a similar trend to that observed in Masson staining (Figure S12, Supporting Information). Together, the efficient photoprotection on the skin against photodamage has been roundly confirmed with an in vivo model, and the protective mechanism will be subsequently evaluated.

### 2.5 In Vitro and In Vivo Genotoxicity Evaluation

**Figure 5:**
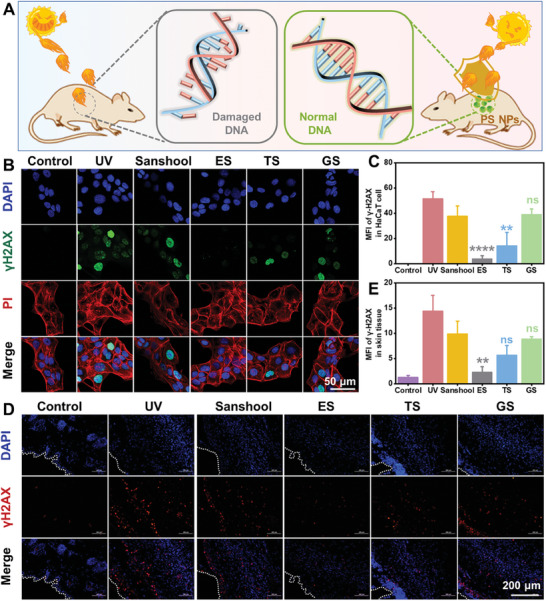
In vitro and in vivo genotoxicity Evaluation. A) The schematic illustration of PS NPs protective effects on genotoxicity. B) γH2AX immunofluorescence staining images and C) quantitative analysis of photodamaged HaCaT cells with different treatments. D) γH2AX immunofluorescence staining images and E) quantitative analysis of photodamaged skin tissues with different treatments. The ns represents no significant difference; ^*^ represents *p* < 0.05; ^**^ represents *p* < 0.01; ^***^ represents *p* < 0.001; and ^****^ represents *p* < 0.0001 versus sanshool group.

The photodamage would introduce severe genotoxicity on HaCaT cells and trigger a cascade of irreversible damage. (**Figure** **5**A) Thus, γH2AX immunofluorescence was employed to evaluate the protective mechanism of PS NPs on skin tissues.^[^ ^43^ ^]^ The occurrence of DNA double‐strand breaks (DSBs) is widely recognized as a perilous issue that can have severe repercussions on cell viability.^[^ ^44^ ^]^ These breaks are typically a result of the interaction between cellular DNA and ROS that are activated by UV. Chromatin‐associated DSBs trigger the prompt initiation of phosphorylation in a specific variant of histone H2A called H2AX, leading to the production of γH2AX.^[^ ^45^, ^46^ ^]^ As shown in Figure 5B,C and Figure S13 (Supporting Information), a notable and statistically significant rise in the quantity of γH2AX foci was detected when comparing HaCaT cells exposed to radiation with control cells. Pre‐treated HaCaT cells with sanshool exhibited a relatively weaker fluorescence intensity of γH2AX compared to cells subjected solely to UV irradiation. However, there was no significant difference in the number of γH2AX foci between the GS group and the sanshool group. Importantly, a significant reduction in the number of γH2AX foci was observed in HaCaT cells treated with ES and TS NPs, relative to the sanshool group. Specifically, the γH2AX fluorescence intensity in HaCaT cells treated with ES NPs approached that of non‐UV irradiated cells, indicating that ES has a superior ability compared to sanshool in mitigating DNA damage and genotoxicity induced by UV radiation.

Furthermore, we utilized immunofluorescence verification to confirm the expression of γH2AX in the skin tissue of UV‐exposed mice. After sacrificing the mice on the fifth day, the back skin was embedded, stained, and subsequently observed for fluorescence expression under a microscope. Consistent with the cellular fluorescence findings, the skin tissue not subjected to UV radiation exhibited minimal γH2AX fluorescence expression (Figure 5D,E; Figure S14, Supporting Information). Following continuous UV exposure, both the epidermis and dermis of the mouse skin displayed an increased presence of γH2AX fluorescence foci (UV group). Pre‐treatment with sanshool exhibited a partial reduction in γH2AX expression, yet the fluorescence intensity remained relatively elevated compared to normal tissue. Among all the groups treated with PS NPs, the ES group demonstrated a notable decrease in fluorescence intensity within the mouse skin tissue, as compared to the sanshool group, aligning with the results of cellular fluorescence (Figure 5D,E; Figure S14, Supporting Information). Based on all the in vitro and in vivo biological validation results mentioned above, it could be concluded that the fabrication of PS NPs could improve the photoprotective effects on skin against photodamage, and ES NPs performed better due to the superior photo absorbance and antioxidative abilities.

## 3 Conclusion

In summary, a series of NPs with uniform sizes, including ES, TS, and GS NPs, have been fabricated via boron ester reactions and noncovalent interactions between natural polyphenols and phenylboronic acid group‐modified sanshool. The NPs structures and reaction mechanism were also evaluated. Interestingly, the natural polyphenols have been shown to assist in improving key properties of sanshool, such as photo absorbance and antioxidative abilities, as well as their corresponding stabilities. Furthermore, the intracellular and in vivo models have been established to demonstrate the enhanced photoprotective effects of PS NPs compared to naked sanshool. This improvement could be attributed to their excellent biocompatibility, efficient free radical scavenging, down‐regulated apoptosis ratios, and reduced genotoxicity. Overall, it could be proposed that natural polyphenols can serve as an efficient platform for enhancing the vital properties of natural active molecules.

## 4 Experimental Section

### Fabrication of NPs

The PS NPs were prepared with sanshool‐boric acid and a series of natural polyphenols. Specifically, the sanshool‐boric acid solution was first prepared (25 mg mL^−1^). 20 mL natural polyphenol aqueous solutions were respectively prepared including EGCG (0.75 mg mL^−1^), tea polyphenol (1.00 mg mL^−1^), and grape seed polyphenol (1.00 mg mL^−1^), and the above solution was stirred for 30 utes. After that, 1 mL sanshool‐boric acid solution was dropwise added into each polyphenol solution and the mixtures were stirred for another 12 h. The PS NPs were obtained via centrifugation (15 000 rpm, 8 min) and washing three times, and were labelled as ES, TS, and GS NPs, respectively.

### Cell Culture

The immortalized human keratinocyte cell line (HaCaT) was acquired from the Cell Resource Center at Peking Union Medical College in China. These cells were cultured in MEM supplemented with 10% (v/v) fetal bovine serum (FBS) and 1% (v/v) penicillin/streptomycin. The culture conditions involved maintaining the cells at 37 °C in an environment consisting of 5% CO_2_ and 95% air.

### UV Irradiation of HaCaT Cells

To investigate the effects of UV irradiation, the cells were exposed to a dose of 80 mJ cm^−2^ of UVB (311 nm, obtained from Sigma, China). The UV radiometer (Photoelectric Instrument, Beijing Normal University, China) measured a fixed position's UVB radiation intensity to be 0.825 mW cm^−2^. To calculate the UVB dose (mJ cm^−2^), the UVB radiation intensity (mW cm^−2^) is multiplied by the time (s). As a result, an irradiation time of 96 s was necessary to achieve an 80 mJ cm^−2^ dose of UVB.

### Analysis of Cell Viability

CCK‐8 assay (CCK‐8; Dojindo, Kumamoto, Japan) was utilized to assess the impact of varying concentrations of PS NPs on the viability of HaCaT cells. The findings from the cell viability analysis indicated that the most effective concentration for photoprotection with PS NPs was 2.5 mg L^−1^. Consequently, this condition was employed to treat cells in subsequent in vitro evaluation.

### Intracellular ROS Scavenging by Polyphenol‐Sanshool

HaCaT cells were cultured in 6‐well plates at a density of 180 000 cells per well. They were then treated with varying materials of PS NPs for 24 h, followed by exposure to UV. Subsequently, the cells were stained with DCFH‐DA and Hoechst 3325 in the dark at 37 °C for 10 min each. The outcomes were captured using a laser scanning a confocal microscope and analyzed using ImageJ. The determination of SOD levels was conducted following the instructions provided by the assay kit manufacturer.

### Cell Apoptosis

Following the treatments, PBS was used to wash all six groups of cells. Subsequently, the cells were suspended in a Binding Buffer. Annexin V (5 µL) and propidium iodide (5 µL) (Annexin V‐FITC/PI apoptosis kit; KeyGen Biotech, China) were added to the cell suspension. The levels of early, late, and total apoptosis were detected and recorded using a flow cytometer (CytoFLEX, Beckman Coulter, Inc., USA). The percentage of total apoptotic cells was calculated and subjected to statistical analysis.

### Animal

All animal experiments in this work were approved by the ethics committee of West China Hospital, Sichuan University (Approval number: 20220223116), and carried out following the ARRIVE guidelines. The mice were randomly assigned to six groups, which consisted of: the control group (no treatment), the UV group (blank solvent and UV irradiation), the ES group (pretreated with ES and exposed to UV), the TS group (treated with TS prior to UV radiation), the GS group (received GS and UV irradiation), and the sanshool group (treated with sanshool and exposed to UV). The UV intensity was measured at 1000 µW cm^−2^, and the total energy delivered during the irradiation process was 1.5 J cm^−2^. This was achieved by subjecting the cells to irradiation for a duration of 300 s each day, over a period of 5 consecutive days.

### Animal Skin Appearance

On a daily basis, the skin on the mice's back was captured using a camera, and the assessment of skin lesions was carried out by evaluating the photodamage score (0: Flesh‐colored skin, smooth and plump; 0.5: Flesh‐colored skin, slightly rough; 1: Red patches, moderately rough skin, deepened skin lines; 1.5: Red patches, noticeably rough skin, minor flaking, some wrinkles; 2: Purple‐red ulcerative condition, significantly rough and thickened skin, extensive flaking, increased wrinkles) and measuring the skin‐fold thickness, following the methodology outlined in the previously published study. The measurements were consistently performed by the same operator, who repeated the process three times to get the average value.

### Histological Assessment

Following 5 days of UV irradiation, skin samples from mice were obtained for histological assessment. H&E, Masson, as well as EVG stains were applied to all samples. The sections were documented using optical microscopy and assessed by two experienced investigators who were blinded to the experimental conditions.

### Immunofluorescence of γH2Ax

To conduct cellular immunofluorescence staining, a specific monoclonal antibody targeting Ser139‐phosphorylated H2Ax (γH2Ax) (Cell Signaling Technology) was employed. HaCaT cells were initially seeded on coverslips in 12‐well plates, followed by UVB irradiation on the subsequent day. After 4 h of treatment, the cells were fixed in 4% formaldehyde (room temperature, 15 min). Subsequently, blocking was performed using 5% normal goat serum (60 min). The cells were then incubated overnight at 4 °C with the γH2Ax antibody (1:400 in 1% BSA). Following that, a fluorochrome‐conjugated anti‐rabbit secondary antibody (Cell Signaling Technology), diluted with a ratio of 1:1000, was incubated at room temperature for 1 h. Phalloidin (PI, from Cell Signaling Technology) was used to stain actin filaments which helps to further determine the expression location of γ‐H2AX, and the detailed condition was 15 min at room temperature. Coverslips were mounted using ProLong Gold Antifade Reagent with DAPI (Cell Signaling Technology) overnight. Immunofluorescence images were obtained using a confocal microscope. The quantification of fluorescent cells expressing γH2Ax was performed using ImageJ software.

### Statistical Analysis

Student t‐tests and one‐ or two‐way ANOVA tests were performed and statistical plots were drawn using GraphPad Prism 9 and Origin. The significance of multiple comparisons was tested using Tukey's or Dunnett's multiple comparison tests. And *p* value <0.05 was considered a statistically significant difference.

## Conflict of Interest

The authors declare no conflict of interest.

## Supporting information

Supporting Information

## Data Availability

The data that support the findings of this study are available from the corresponding author upon reasonable request.
