# Supplementary material for: Polyphenolic Platform Ameliorated Sanshool for Skin Photoprotection
Source: Adv Sci (Weinh). 2024 Feb 15;11(16):2310012. doi: 10.1002/advs.202310012 (PMC11040382; doi:10.1002/advs.202310012)
Supplement: Supplementary file 1 — Supporting Information [file ADVS-11-2310012-s001.pdf]

ADVANCED SCIENCE
Open Access

Supporting Information

for *Adv. Sci.*, DOI 10.1002/advs.202310012

Polyphenolic Platform Ameliorated Sanshool for Skin Photoprotection

Tianyou Wang, Linghong Guo, Shuwei Wu, Yuanyuan Xu, Junmei Song, Yi Yang, Hengjie Zhang, Dongcui Li, Yiwen Li, Xian Jiang and Zhipeng Gu**

Supporting Information

Polyphenolic platform ameliorated sanshool for skin photoprotection

Tianyou Wang, Linghong Guo, Shuwei Wu, Yuanyuan Xu, Junmei Song, Yi Yang, Hengjie Zhang, Dongcui Li, Yiwen Li, Xian Jiang and Zhipeng Gu**

Materials

Epigallocatechin gallate (EGCG, DASF Bio-Technology, 98%), Tea polyphenol (mainly including epicatechin, epigallocatechin, EGCG and epicatechin gallate, Wuxi Taiyo Green Power, 95%), Grape seed polyphenol (Oligomeric Proanthocyanidins, including catechins of different degrees of polymerization from 2~5, DASF Bio-Technology, 98%), 4-Carboxylphenylboronic acid pinacol ester (Energy chemical, 98%), (2E, 6Z, 8E, 10E)-N-(2-hydroxy-2-methylpropyl)dodeca-2,6,8,10-tetraenamide (Hydroxy-α-Sanshool, DASF Bio-Technology, 98%), 4-Dimethylaminopyridine (DMAP, J&K Scientific, 99%), Rhodamine B (Energy chemical, 95%), N,N'-Diisopropylcarbodiimide (DIPC, J&K Scientific, 99%), 2,2-diphenyl-1-picrylhydrazyl (DPPH, Alfa Aesar, 95%), 2,2'-azino-bis (3-ethylbenzothiazoline-6-sulfonic acid) diammonium salt (ABTS, TCI, 98%), Hydrochloric acid (Kelong chemical, AR), Dimethyl sulfoxide (DMSO, Kelong chemical, AR), Dichloromethane (DCM, Kelong chemical, reagent grade), N, N-Dimethylformamide (DMF, Kelong chemical, AR), Ethanol (Kelong chemical, reagent grade), Pentobarbital sodium (Kelong chemical, AR), Paraformaldehyde (Sigma, reagent grade), DCFH-DA (Sigma), Ser139-phosphorylated H2Ax (γH2Ax) (Cell Signaling Technology), .Phalloidin (Cell Signaling Technology). All chemicals were used without further purification.

Synthesis of pheylboric acid group modified sanshool

Briefly, pheylboric acid group modified sanshool was synthesized with sanshool and 4-Carboxylphenylboronic acid pinacol ester by esterification reaction. Briefly, 52.7 mg (0.20 mmol) sanshool and 59.5 mg (0.24 mmol) 4-Carboxylphenylboronic acid pinacol ester were fully dissolved in 4 mL dichloromethane (DCM). Then, 14.7 mg (0.12 mmol) 4-Dimethylaminopyridine (DMAP) and 125 μL (0.80 mmol) N, N'-Diisopropylcarbodiimide (DIPC) were added into above solution and mild stirring was kept for 24 h. The DCM was evaporated under vacuum and N, N-Dimethylformamide (DMF) was utilized to redissolve the

crude products. Then, 1 M hydrochloric acid was utilized for deprotection and the diethyl ether was used for further precipitation. The yellowish solid products were obtained under vacuum to remove solvent.

Photoprotective abilities and corresponding stabilities

The UV–Vis spectra were first employed with PerkinElmer Lambda 650 UV/Vis spectrophotometer (the slit was 2 nm). The simulated sunlight (2 W, 2 h, CEL-PE300L-3A) was employed to evaluate the stabilities of PS NPs, and the absorbance at 271 nm was utilized for the retention rates calculation. For the photoprotective experiments for Rh B, the Rh B solution was prepared with a concentration of 10 μg/mL. On the other hand, the PS NPs loaded filter papers were also prepared (blank: deionized water; sanshool, ES, TS and GS NPs with the amount of 2 mg), and stored for various time up to five days. Subsequently, the Rh B solution was exposed to simulated sunlight (2 W, 2 h) with different treatments. The formula A_t/ A_0 × 100% was used to assess the photostability, where the A0 was the initial absorbance of Rh B solution at 564 nm and A_t was the absorbance of Rh B solution at 564 nm with different treatments.

DPPH assay and corresponding antioxidative stability

The DPPH assay was carried out for antioxidative ability evaluation. Briefly, DPPH solution was prepared freshly with the concentration of 1.0 mM in ethanol and sample solutions (sanshool and PS NPs, 0.6 mg/mL) were also prepared, respectively. Then, 300 μL DPPH solution and 100 μL samples were added into 2600 μL ethanol, and corresponding absorbance at 517 nm was recorded with different time. Furthermore, the antioxidative stability of above samples were tested, and the original antioxidative abilities were recorded as A0. The simulated sunlight (2 W, CEL-PE300L-3A) was applied onto each group for different time, and the antioxidative abilities were recorded as At. The retention ratio was calculated as At/ A0*100%. For the long-term antioxidative stability assay, the samples were placed in outdoor environment for various time up to five days, and the scavenging abilities were respectively tested.

ABTS assay and corresponding antioxidative stability

The ABTS assay was carried out for antioxidative ability evaluation. Briefly, 54.04 mg ABTS was dissolved in deionized water and 9.93 mg potassium peroxodisulfate was subsequently added. Then, the solution was mildly stirred at room temperature with a dark atmosphere overnight. Sample solutions (sanshool and PS NPs, 0.6 mg/mL) were also prepared for further testing. Then, 100 μL ABTS solution and 100 μL samples were added into 2800 μL deionized water, and corresponding absorbance at 734 nm was recorded with different time. Furthermore, the antioxidative stability of above samples were tested, and the original antioxidative abilities

were recorded as A0. The simulated sunlight (2 W, CEL-PE300L-3A) was applied onto each group for different time, and the antioxidative abilities were recorded as At. The retention ratio was calculated as At/ A0*100%. For the long-term antioxidative stability assay, the samples were placed in outdoor environment for various time up to five days, and the scavenging abilities were respectively tested.

Characterization

^{1}H nuclear magnetic resonance (NMR) spectra were performed in DMSO-d6 (Aldrich, 99.8 % D) utilizing a Bruker AV III HD 400 MHz NMR spectrometer. The ^{1}H NMR spectra were referenced to the residual proton signals in DMSO-d6 at δ 2.50 ppm. Phenom Pro microscope was used for obtaining scanning Electron Microscope (SEM) images. Aqueous solutions of materials (1 mg/mL) were settled onto the substrate of mica, and then rotated to remove the excess water. The prepared samples were fully dried before testing. Malvern Nano ZS ZEN3690 instrument was used for obtaining Hydrodynamic Diameter (DH) as well as Zeta Potential (ZP). The results were averaged through 12 runs for 3 times in deionized water (50 μg/mL). PerkinElmer Lambda 650 UV/Vis spectrophotometer was used for UV-vis spectra with slit of 2 nm. PHI Quantera SXM spectrometer using Al Ka radiation and spectra was used for X-ray photoelectron spectroscopy (XPS) at the pass energy of 160 eV for survey spectra and 20 eV high-resolution spectra of C 1s, O 1s, N 1s, B 1s regions with the 300 ms dwell time. Electrospray ionization mass spectrometry (ESI-MS) spectrum was performed on the Applied Biosystems Biosystems API 2000 with cationic mode electrospray ionization. The samples solution was injected at a flow rate of 10 μL/min and the spray voltage was 5 kV. The pressure of auxiliary and sheath was 45 psi.

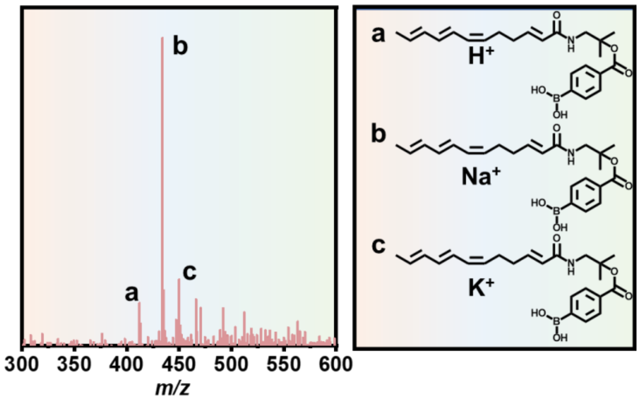

Figure S1. The ESI-MS spectrum and proposed structures of pheylboric acid group modified sanshool.

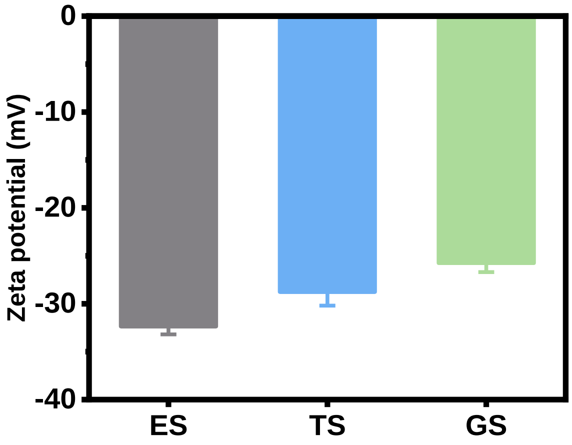

Figure S2. The zeta potential of ES, TS and GS NPs, respectively.

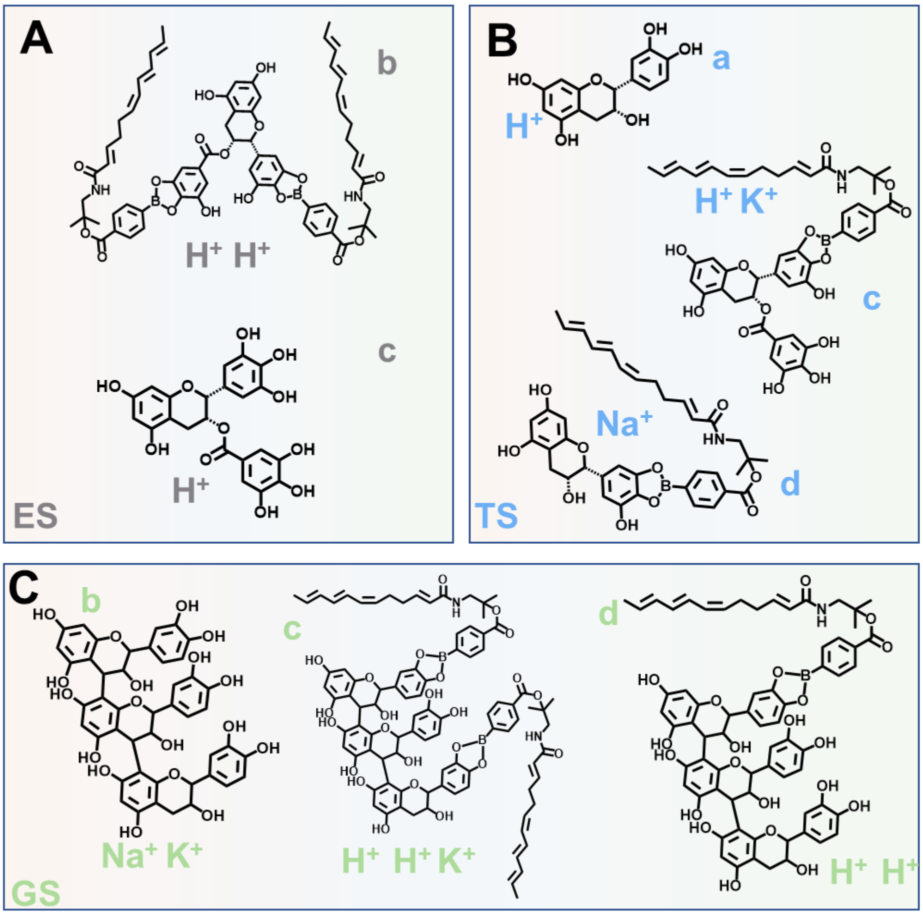

Figure S3. The proposed structures which were assigned to the peaks in Figure 1G-I of (A) ES, (B) TS and (C) GS, respectively.

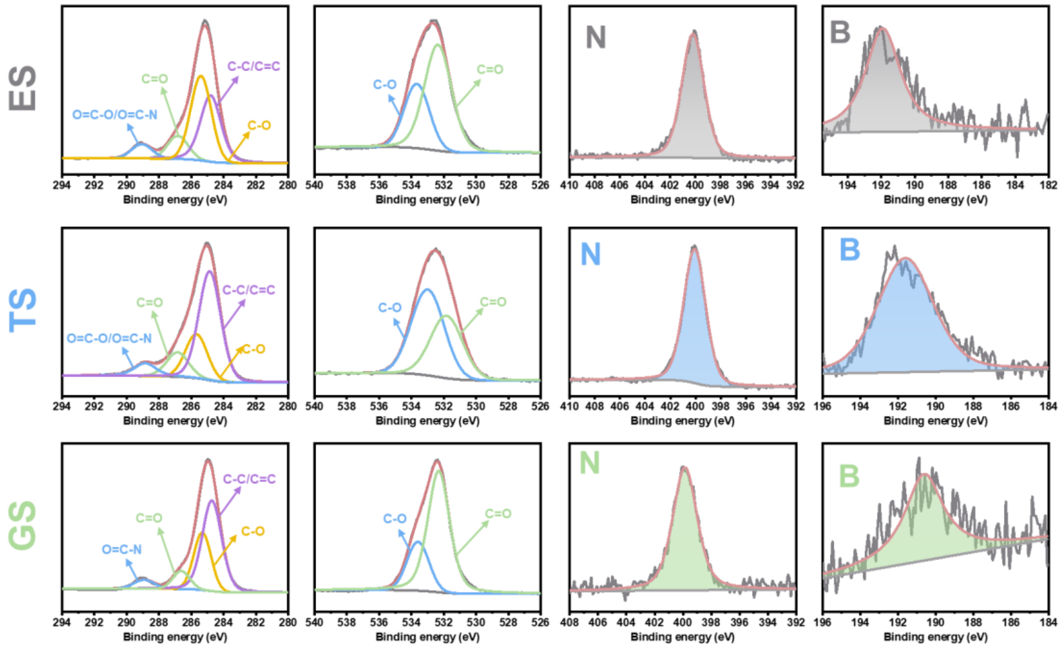

Figure S4. The C 1s, O 1s, N 1s and B 1s XPS spectra of ES, TS and GS NPs, respectively.

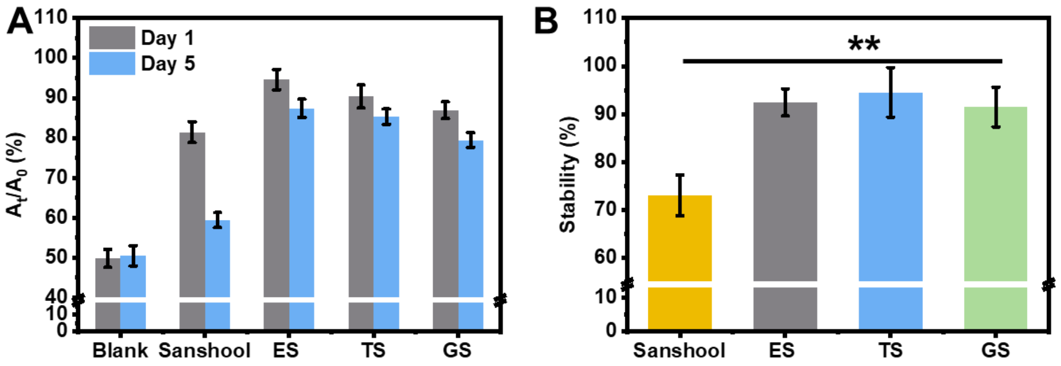

Figure S5. The photoprotective effects and stability towards Rh B of blank, sanshool, ES, TS and GS NPs, respectively. The ns represents no significant difference; * represents $p < 0.05$; ** represents $p < 0.01$; *** represents $p < 0.001$; and **** represents $p < 0.0001$.

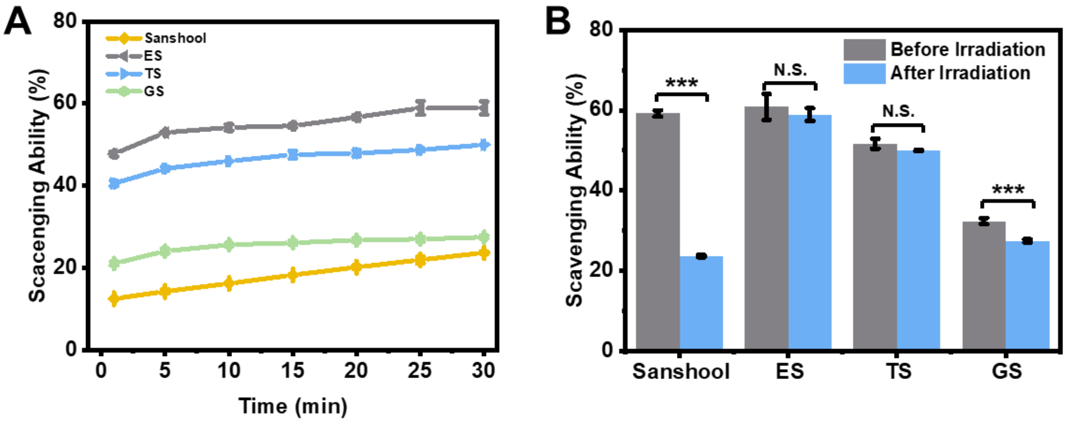

Figure S6. The (A) scavenging abilities after simulated sunlight irradiation and (B) stabilities of sanshool, ES, TS and GS NPs towards DPPH free radicals. The ns represents no significant difference; * represents $p < 0.05$; ** represents $p < 0.01$; *** represents $p < 0.001$; and **** represents $p < 0.0001$.

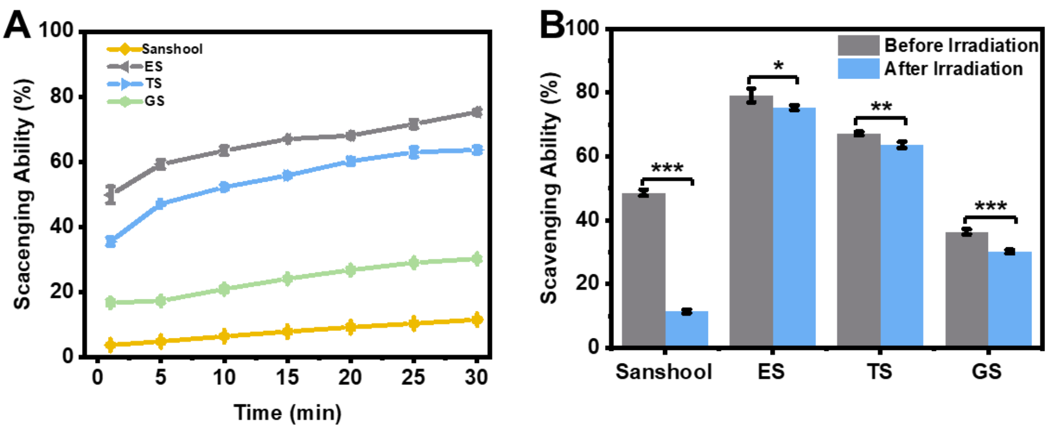

Figure S7. The (A) scavenging abilities after simulated sunlight irradiation and (B) stabilities of sanshool, ES, TS and GS NPs towards ABTS free radicals. The ns represents no significant difference; * represents p < 0.05; ** represents p < 0.01; *** represents p < 0.001; and **** represents p < 0.0001.

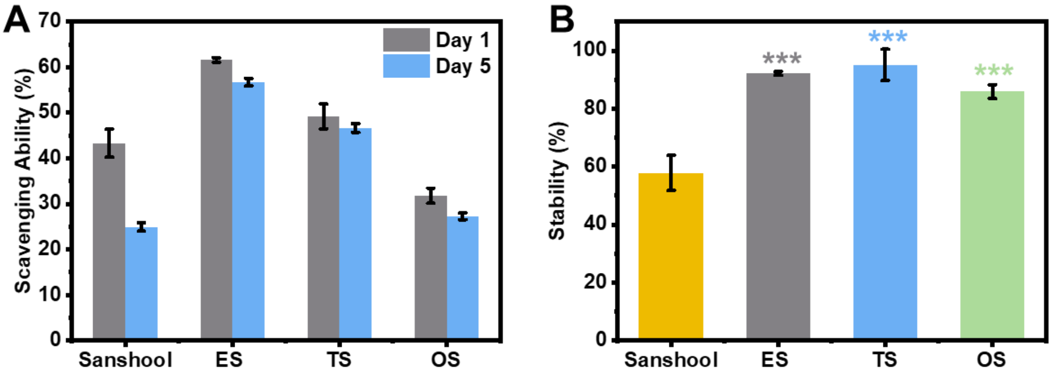

Figure S8. The long-term antioxidative stabilities of sanshool, ES, TS and GS NPs towards DPPH free radicals. The ns represents no significant difference; * represents p < 0.05; ** represents p < 0.01; *** represents p < 0.001; and **** represents p < 0.0001 *vs.* sanshool group.

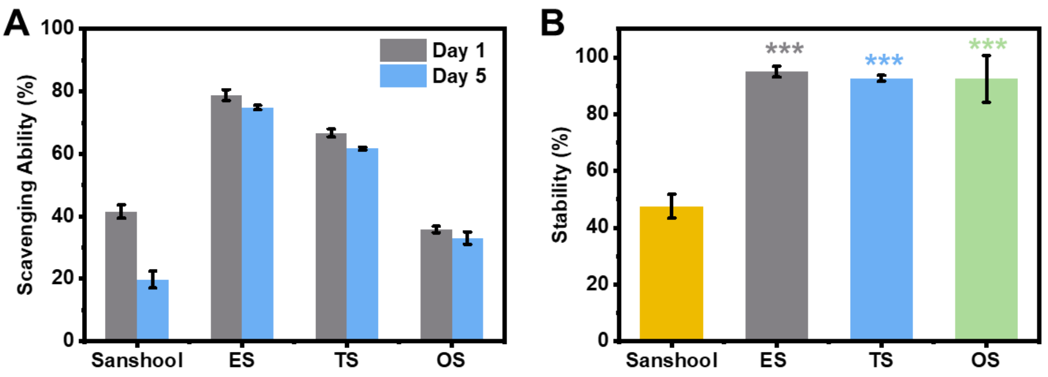

Figure S9. The long-term antioxidative stabilities of sanshool, ES, TS and GS NPs towards ABTS free radicals. The ns represents no significant difference; * represents p < 0.05; ** represents p < 0.01; *** represents p < 0.001; and **** represents p < 0.0001 *vs.* sanshool group.

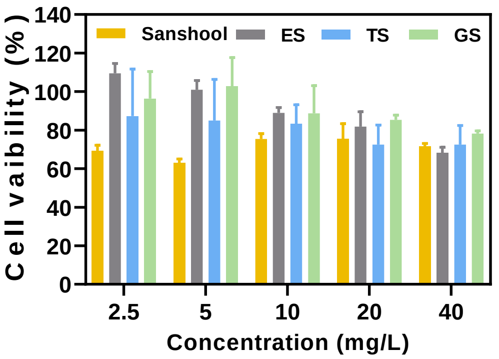

Figure S10. The cell viabilities treated with sanshool, ES, TS and GS NPs at different concentrations, respectively.

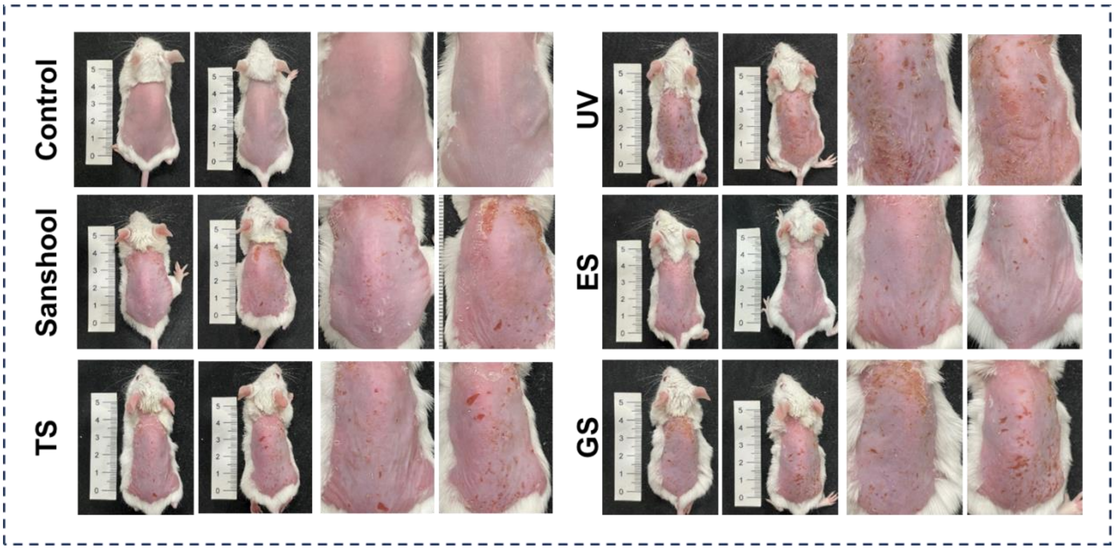

Figure S11. The optical images of skin with different treatments after UV irradiation. (Parallel experimental groups)

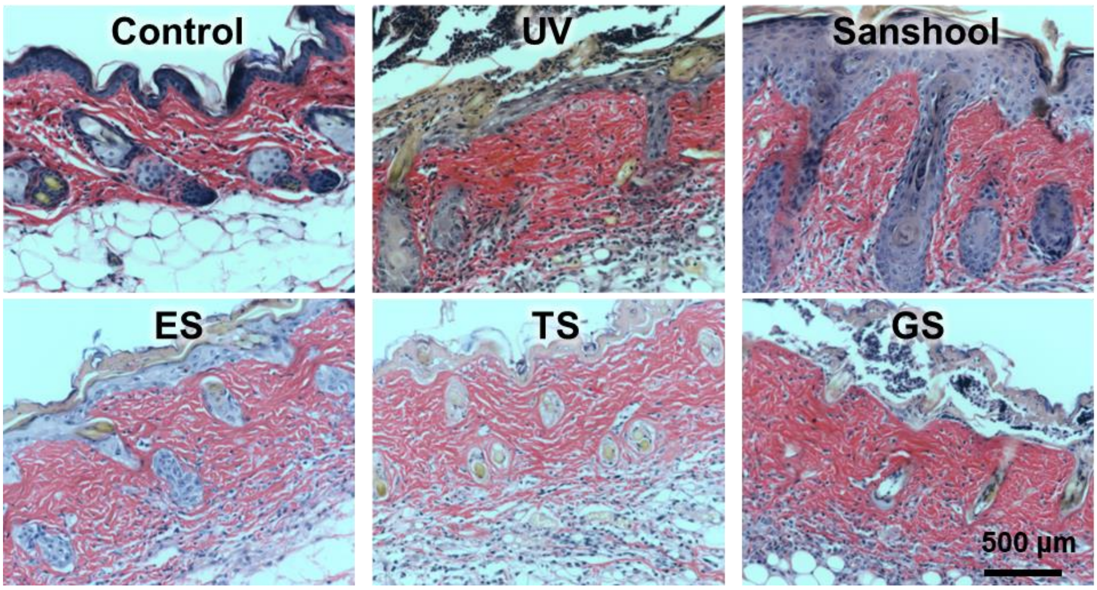

Figure S12. The EVG staining images of mice skin in each group.

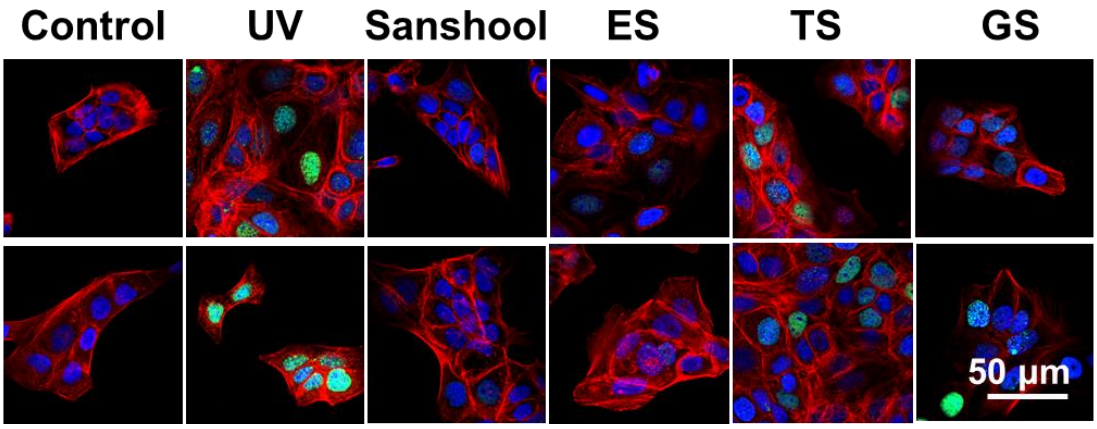

Figure S13. (A) γH2AX immunofluorescence staining images of photodamaged HaCaT cells with different treatments. (Parallel experimental groups)

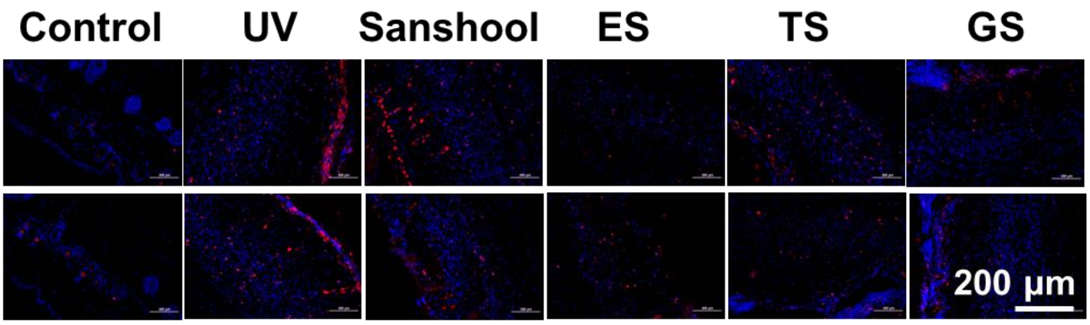

Figure S14. (A) γH2AX immunofluorescence staining images of photodamaged skin tissues with different treatments. (Parallel experimental groups)
